# PRUNE1 and NME/NDPK family proteins influence energy metabolism and signaling in cancer metastases

**DOI:** 10.1007/s10555-023-10165-4

**Published:** 2024-01-05

**Authors:** Veronica Ferrucci, Santosh Lomada, Thomas Wieland, Massimo Zollo

**Affiliations:** 1 Department of Molecular Medicine and Medical Biotechnology, DMMBM, University of Naples, Federico II, Via Pansini 5, 80131 Naples, Italy; 2 CEINGE Biotecnologie Avanzate “Franco Salvatore”, Via Gaetano Salvatore 486, 80145 Naples, Italy; 3 Experimental Pharmacology Mannheim, European Center for Angioscience, Medical Faculty Mannheim, Heidelberg University, 68167 Mannheim, Germany; 4 DZHK, German Center for Cardiovascular Research, Partner Site Heidelberg/Mannheim, 68167 Mannheim, Germany; 5 Medical Faculty Mannheim, Ludolf Krehl-Str. 13-17, 68167 Mannheim, Germany; 6 DAI Medicina di Laboratorio e Trasfusionale, ‘AOU’ Federico II Policlinico, 80131 Naples, Italy

**Keywords:** PRUNE1, Exopolyphosphatase, NME family, Nucleoside diphosphate kinase NDPK, NMR structures, Energy metabolism, Tumor microenvironment, Immune system

## Abstract

We describe here the molecular basis of the complex formation of PRUNE1 with the tumor metastasis suppressors NME1 and NME2, two isoforms appertaining to the nucleoside diphosphate kinase (NDPK) enzyme family, and how this complex regulates signaling the immune system and energy metabolism, thereby shaping the tumor microenvironment (TME). Disrupting the interaction between NME1/2 and PRUNE1, as suggested, holds the potential to be an excellent therapeutic target for the treatment of cancer and the inhibition of metastasis dissemination. Furthermore, we postulate an interaction and regulation of the other Class I NME proteins, NME3 and NME4 proteins, with PRUNE1 and discuss potential functions. Class I NME1–4 proteins are NTP/NDP transphosphorylases required for balancing the intracellular pools of nucleotide diphosphates and triphosphates. They regulate different cellular functions by interacting with a large variety of other proteins, and in cancer and metastasis processes, they can exert pro- and anti-oncogenic properties depending on the cellular context. In this review, we therefore additionally discuss general aspects of class1 NME and PRUNE1 molecular structures as well as their posttranslational modifications and subcellular localization. The current knowledge on the contributions of PRUNE1 as well as NME proteins to signaling cascades is summarized with a special regard to cancer and metastasis.

## Introduction

The human PRUNE1 protein belongs to the DHH (Asp-His-His) protein superfamily and possesses a nucleotide phosphodiesterase (PDE) [1] and an exopolyphosphatase (exopolyphosphatase/pyrophosphatase, PPX/PPase) function (the latest with four orders of magnitude higher), with higher affinity for short-chain over long-chain inorganic polyphosphates (polyPs) [2].

PRUNE1 is a naturally unfolded protein with the ability to interact with several binding partners of which the most important are NME1 and NME2 [3]. Other known PRUNE1 protein interaction partners are GSK-3β [4], ASAP1 [5], and microtubules (MTs) [6]. Because of its both enzymatic activities and the variety of interactors, PRUNE1 can modulate several signaling cascades, such as Wnt [4], TGF-β [7], MAPK/ERK [8], and the epithelial–mesenchymal transition (EMT) processes [7].

Here, we discuss how PRUNE1/NME1/2 regulation might influence energy metabolism and signaling in cancer metastases. The NME/NDPK (Nucleoside Diphosphate Kinase) family of proteins are a group of enzymes that play crucial roles in cellular metabolism in various cellular processes and have been found in a wide range of organisms [9]. These proteins are highly conserved, and their functions and evolution have been extensively studied. They are involved in phosphoryl transfer reactions, primarily catalyzing the transfer of phosphate groups between nucleoside diphosphates (NDPs) and nucleoside triphosphates (NTPs) [10]. This activity is essential for maintaining optimal intracellular nucleotide pools and regulating nucleotide-dependent processes. Thus, the primary function of NME proteins “intrinsic” is to regulate the cellular levels of nucleoside triphosphates, such as ATP and GTP, which are the main energy currency and signaling molecules in cells. By converting NDPs and dNDPs to NTPs and dNTPs, NME proteins participate in various biological processes that require energy, including DNA replication [11], RNA transcription [12], protein synthesis, and cellular differentiation [13]. Their secondary activity “extrinsic” is mainly involved in signal transduction processes, in which NME proteins directly interact with specific cellular proteins and modulate their activities [1]. They are involved in several processes, such as cell proliferation, differentiation, and apoptosis. Of importance, other NME functions have been identified regulating cellular complex via its enzymatic activities (i.e., NDPK, histidine kinase, and 3′–5′ exonuclease) and the isolation of other NME1 isoforms (i.e., NME2-4) with distinct subcellular localization patterns consequently suggest several other signaling pathways involved. In conclusion, by controlling the cellular nucleotide pool, NME proteins are involved in various processes including DNA replication and repair, RNA processing, cell proliferation, cell motility, signal transduction, energy metabolism, cytoskeleton modeling, and microtubule dynamics.

PRUNE1 has been reported to be overexpressed and positively correlated with disease progression and metastatic status in several tumors, including medulloblastoma (MB) [7], neuroblastoma (NB) [14], thyroid cancer [15], breast cancer (BC) [1, 8]), non-small cell lung (NSCL) cancer [4], esophageal squamous cell carcinoma [16], gastric cancer [17], colorectal cancer [18], and multiple myeloma [19]. Additionally, NME family proteins have been investigated in several solid tumor types (e.g., melanoma, breast, colon, lung, liver, ovary, prostate, and oral and hepatocellular carcinoma); an inverse correlation between NME1 expression and metastatic dissemination has been reported, thus strongly indicating an anti-metastatic activity [20–22]. On the other hand, in certain tumor types (e.g., NB [14], MB [7], hematopoietic malignancies and osteosarcoma [23]), the high expression of NME1 is often correlated with metastatic status and poor patient outcome, thus postulating an additional level of regulation in such tumors may be because of its multi-faceted roles in regulating complex cellular via its enzymatic activities, as previously reported.

It is noteworthy that unbalancing the PRUNE1/NME1 interaction causes a switch to pathological conditions (i.e*.*, cancer) via affecting several protein networks [1, 24, 25]. Overall, the PRUNE1/NME1 protein complex may explain the dual role of NME1 acting as both suppressor and promoter of cancer metastasis. Here we mainly focus on the isoforms NME1-4 together with PRUNE1 and their regulatory functions for those processes relevant to cancer and metastasis.

Nevertheless, the interaction of NME proteins with a variety of protein binding partners is likely crucial to their controversial roles in the context of metastasis niche formation. Indeed, the interaction of NME1 and NME2 with cytoskeletal components (e.g., MT), small GTPases, G protein coupled receptors (GPCRs), ion channels, extracellular matrix, membrane proteins [26], and oncoproteins has been described.

Focusing on metastasis-related proteins and PRUNE1 [7, 14], which also modulate signaling pathways (e.g., TGF-β [7]), we can assign an important function of the protein complex (PRUNE1/NME1-or NME2) for the metabolic reprogramming and the inflammatory communications between tumorigenic and immune cells with actions in TME, thus enhancing cancer and the organization of new metastatic niches.

## PRUNE1/NME1 interaction conserved from *Drosophila melanogaster* to amphibians and mammals

The interaction between PRUNE1 and NMEs was first shown by genetic analyses in *Drosophila melanogaster*, where viable homozygous and hemizygous mutations in “Pn” (*D. melanogaster’*s *Prune* homolog) that were responsible for the “prune” eye color became lethal in the presence of a single mutation (i.e., P97S substitution) in the abnormal wing disc gene “Awd” (*D. melanogaster*’s only NME homolog). These *D. melanogaster* mutants died at the second–third larval instar stage due to abnormal development of mesoderm and neuronal cells [27, 28] mostly due to alterations during the switch from GTP to GDP [29].

The first evidence supporting PRUNE1 and NMEs interaction in mammals came from the typical spatiotemporal co-expression patterns of “Prune-M1” and “NM23-M1” (mouse PRUNE1 and NME1 homologs, respectively) in developing murine central nervous system (cortex, hippocampus, midbrain, and cerebellum, from E10.5 to adulthood) [30]. Additionally, “Prune-X1” and “NME-X1” (i.e., *X. Laevis* PRUNE1 and NME1 homologs, respectively) were found co-expressed during retinal development with a role in eye morphogenesis [31]. Later, the binding of PRUNE1 to NME1/2 protein was further validated in co-immunoprecipitation assays in human triple-negative breast cancer (TNBC) cells (i.e., MDA-MB-435) [32].

## Molecular structure of the PRUNE1/NME1 protein complex

**Fig. 1:**
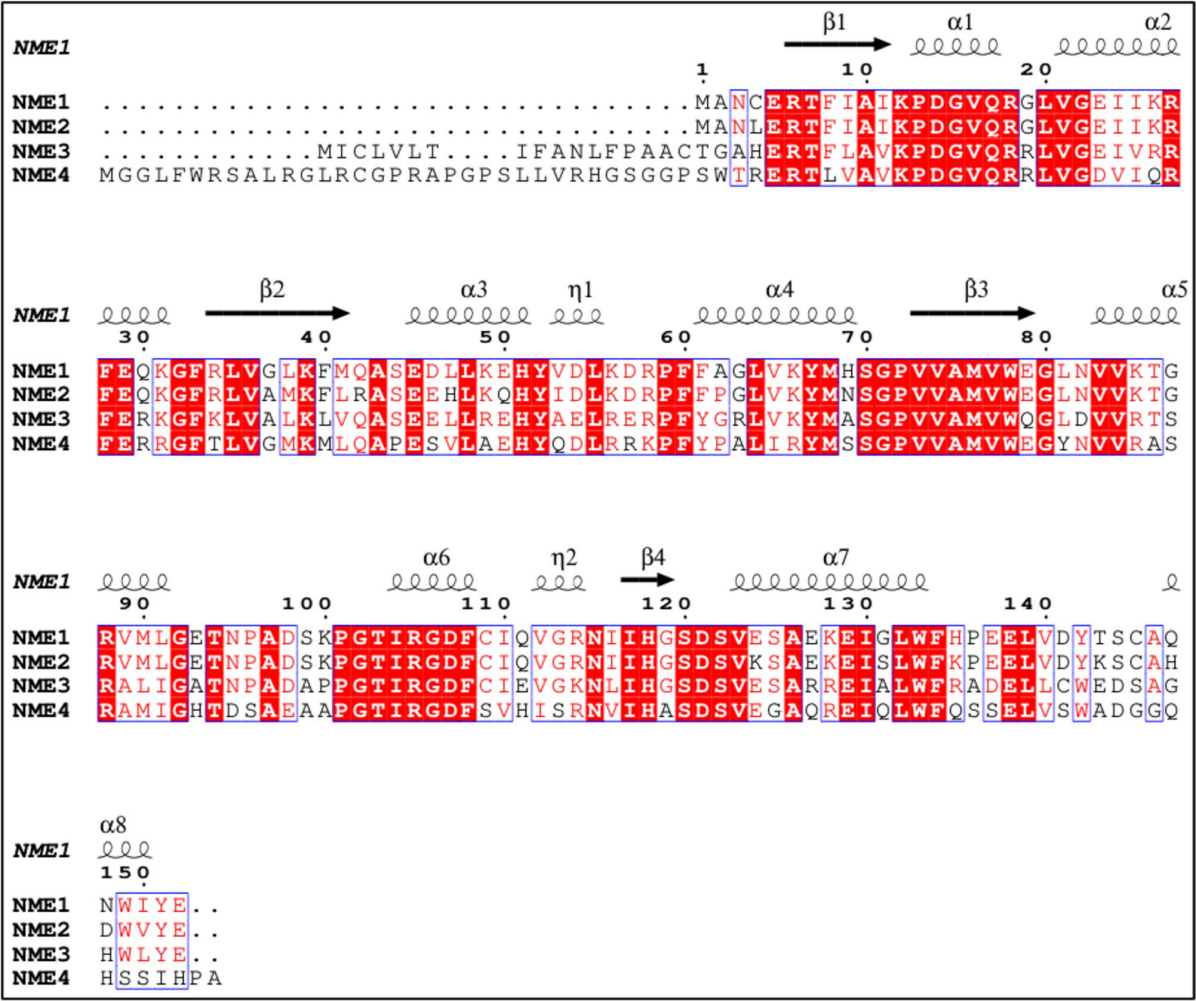
Multiple sequence alignment (MSA) of human NME isoforms. The secondary structure elements for NME1 (PDB:2HVD) are shown. Identical residues are colored white with a red background and similar residues are colored red. MSA was generated using Clustal Omega [38] and rendered using ESPript 3 [39]

Human PRUNE1 is a naturally unfolded protein with the ability to interact with several intracellular binding partners, including NME1 and NME2 [3]. Through structural studies based on nuclear magnetic resonance (NMR), homology modelling, and molecular dynamics, PRUNE1 has been described with two globular domains, an intrinsically disordered proteins (IDP) domain and a small globular region. The Amino-terminus (N-Term) region of the PRUNE1 protein contains two globular domains responsible for its enzymatic functions: the DHH (from 10 to 180 amino acid residues) and DHHA2 (from 215 to 360 amino acids). The Carboxy-terminus (C-term) domain of PRUNE1 (from 354 to 453 amino acid residues) shows low hydrophobicity and a high negative charge, contains part of the DHHA2 domain, and it is mostly unfolded [14]. Of interest, the chemical shift indexing (CSI) obtained on recombinant C-term PRUNE1 protein also suggested the presence of an α-helix secondary structure with three helical stretches spanning from L355 to S365 (α1), from E381 to D388 (α2), and from L428 to Q439 (α3). The small globular region (spanning from amino acids 413 to 439) contains a disulfide bridge crossing C419 and C437 [14]. The C-term domain of PRUNE1 is sufficient for its binding to the C-term of NMEs. Indeed, the interaction region of PRUNE1 involves the IDP domain, and particularly the small globular region (from 387 to 396 amino acid residues), including the α2 helical stretch. In detail, the small globular region in PRUNE1 C-term mediates its binding to NMEs mainly via D388 and D422 amino acid residues [14] that show a high degree of similarity across different species [33]. Furthermore, the single-point mutations P96S and S120G in NME1 that are known to alter its biochemical activities were shown to affect the interaction with PRUNE1 [3]. Of interest, the regions of NME1 and NME2 from their serine 120 (S120) to serine 125 (S125) residues are responsible for the intracellular interaction with PRUNE1 when phosphorylated by casein kinase I and II (CKI and CKII, respectively) [32]. To date, nothing is known about interactions of PRUNE1 with NME3 or NME4 although it could potentially exist because of the similarity of the protein region of interaction between PRUNE1 and NME1 [33], and the other isoforms by observing the 3D protein conformations of C-term domains (α6, β4, α7) are well conserved within all class I isoforms (as further discussed below, see additionally Fig. 1). For the above reasons, we might speculate that a similar interaction between PRUNE1 occurs with homo- and/or hetero-oligomerization NME1-4 isoforms supported by additional preliminary data which show colocalization of PRUNE1 and NME1 protein on mitochondria in human retinal pigment epithelium (RPE) cells (Ferrucci et al. presented these results at the NME 2023 conference; see details in the acknowledgement section). Although such a role of the complex in mitochondria needs further *in vivo* validations, we hypothesize that it might be of importance as a strong activator of energy metabolism within the mitochondria.

Here, below, we will dissect the molecular structure of the NME class I isoforms in the context on how these proteins can potentially be PRUNE1 partners, thus consequently regulating additional cellular signaling and physiology.

## General aspects of NME class 1 molecular structures

Currently, X-ray crystallography has been employed to investigate the molecular structures of human NMEs [34, 35]. Among the class I isoforms, the genes encoding NME1 and NME2 are located on chromosome 17, while the genes encoding the 3 and 4 isoforms are on chromosome 16. NME1 exhibits approximately 88%, 59%, and 44% homology with the NME 2, 3, and 4 isoforms, respectively (see Fig. 1) [36, 37].

The first human NME isoform to be crystallized was NME2. Through molecular displacement, utilizing the existing structures of the *Drosophila* homolog of NMEs, *Awd*, and *Myxococcus xanthus* nucleoside diphosphate (NDP) kinase as references, the NME2 structures at 2 A° (GDP bound) and 2.8 A° (apoprotein) resolution were resolved [40, 41]. The structure of apo human NME1 was published with a resolution of 2.15 A° [42]. The structure of NME3 has been deposited (PDB:1ZS6) but has not been published yet. Indeed, as reported at the NME 2023 conference (see details in the acknowledgement section), the structure of NME3 has recently been solved with different ligands and as apoprotein (S. Lomada, T. Wieland, personal communication). The structure of apo NME4 was published with a 2.4 A° resolution [43]. Moreover, several crystal structures of the class I NME proteins complexed with different ligands were summarized and compared recently [37].

All NME subunits from prokaryotes to eukaryotes have a conserved ferredoxin fold of about 140 amino acids. While the class I human NME isoforms possess a shared catalytic core, they display unique structural characteristics in other regions. The catalytic core is composed of a helix hairpin αA-α2, residues in helices α0 and α3, and the “Kpn loop,” which forms a clamp that stabilizes the base of the ligand [44]. The catalytic site of NME enzymes is highly conserved across isoforms, which includes a histidine residue that acts as a catalytic base and is essential for phosphoryl transfer [37, 44].

**Fig. 2:**
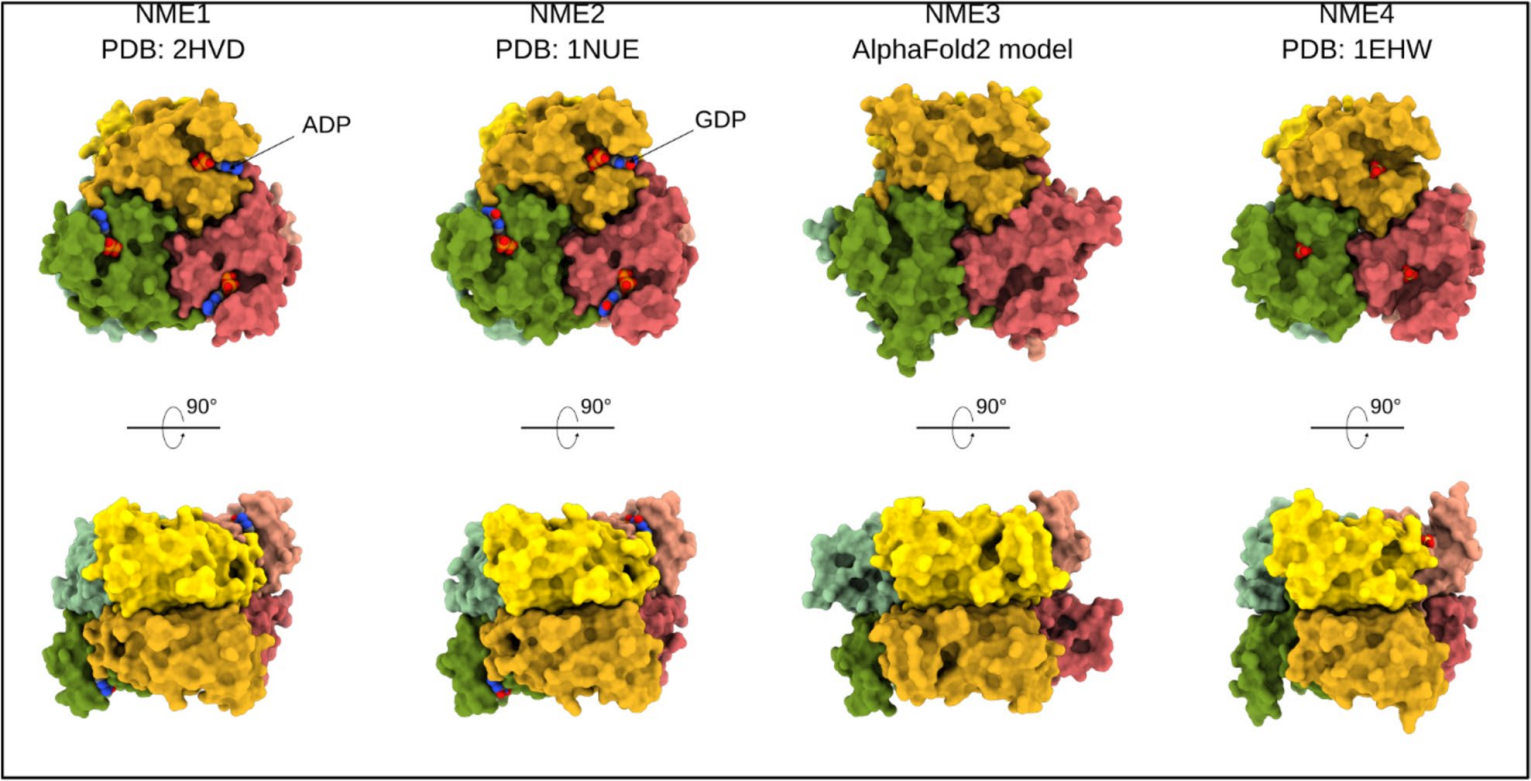
Structures of class I human NMEs, which exist as hexamers. Each monomer is surface rendered and colored uniquely. The ligands are shown as spheres (NME1:ADP and NME2:GDP). Since the experimental structure of NME3 is unavailable, the hexameric NME3 structure was modeled using AlphaFold2 [45]

Class I NME isoforms form hexamers in solution, and within these hexamers, each monomer exhibits phosphotransferase activity. Figure 2 provides a summary of the crystal structures that have been published for NME1, 2, and 4, along with a predicted structure for NME3, which includes the hydrophobic N-terminal amino acids.

The enzymatic reaction involves the transient phosphorylation of the highly conserved catalytic histidine residue from a bound N_1_TP. The formed N_1_DP is replaced by an N_2_DP to which the high energetic phosphate is transferred leading to the formation of N_2_TP [44]. A naturally occurring mutation (S120G) in NME isoforms, with reduced thermal stability, firstly identified in NB [46] is unable to refold in vitro [47, 48]. Interestingly, autophosphorylation at His118 by ATP allows the mutant protein to refold. However, the binding of ADP or a non-hydrolyzable ATP analog, 5'-adenylyl imido-diphosphate, for which the gamma phosphate group cannot be transferred to the histidine residue, does not facilitate refolding. In accordance, a double mutant, H118N/S120G, which is unable of autophosphorylation, remained non-reactivable by ATP, emphasizing the role of S120 in maintaining enzyme stability [49]. Additionally, P96 was found to be crucial for maintaining the quaternary structure stability of the hexamers. Several NME structures indicated that the “kpn loop,” along with the C-term, played a critical role in stabilizing the quaternary structure. Another study proposed that a disulfide cross-linkage between C4 and C145 in NME1 prevented hexamer formation, leading to dissociation. Mutating C145 to Serine (S) stabilized the hexamerization and increased the enzymatic activity, demonstrating the role of a reduced C145 in oligomer formation [50].

Until now, studies analyzing the occurrence of NME hetero-oligomers in vivo are limited and related to the NME1 and NME2 isoforms [51, 52], a truncated version of NME3 with H118N NME1 [53], and a NME2/3 hetero-oligomer [54]. Furthermore, it has been demonstrated that dynamin, a regulator of vesicle fission GTPase, relies on NME1 and NME2 in endocytosis to modulate tumor cell motility and metastasis (detailed discussed in a specific paragraph). Intriguingly, it was revealed that either NME1 or NME2 could promote dynamin oligomerization, and it was suggested that the formation of hetero-oligomers between NME1 and NME2 might influence their protein interactions, subcellular localization, and functions [55].

As discussed above, NME, once phosphorylated by CKI or CKII on S120, S122, and S125, can bind PRUNE1 at level of D388 and D422 amino acid residues located in its C-term globular region [25]. Because the PPX/PPase activity of PRUNE1 has been reported to be inhibited by NME1 [2], a differential composition of NME heterohexamers is of great potential for a link to PRUNE1’s enzymatic activity providing phosphate units (Pi) derived from polyPs degradation to regulate GDP-GTP pools in a particular cellular environment. This has to be further dissected in future investigations.

## PRUNE1 and NMEs subcellular localization and posttranslational modifications

NME proteins are distributed within various subcellular compartments. While class I isoforms are predominantly located in the cytosol and on the plasma membrane, NME4 stands out as an exception, as it possesses a mitochondrial targeting sequence [56]. Furthermore, it has been reported that the additional N-terminal sequence of NME3 likely possesses the capacity to serve as a membrane anchor (see additionally Fig. 2) in the mitochondrial outer membrane, where it plays a crucial role in membrane fusion [57].

PRUNE1 has been mainly reported with a cytoplasmic subcellular localization during interphase and localized at mitotic spindle during mitosis [6]. However, as previously stated, the localization of PRUNE1 at mitochondria might provide an additional level of regulation by protein complex formation with NME1 and the other isoforms.

NMEs have been demonstrated to undergo posttranslational modifications, including phosphorylation [58], acetylation [59], ubiquitination [36], and CoAlation [60], which have the potential to regulate both their enzymatic activity and subcellular distribution. A few examples of posttranslational modifications are summarized below.

Regarding ubiquitination, the SCF-FBXO24 E3 ubiquitin ligase complex was demonstrated to polyubiquitinylate NME1 at K85, along with the two NH2-terminal residues, L55 and K56, to facilitate its proteasomal degradation [61]. Acetylation and deacetylation have been established as regulatory mechanisms for NME4 (also mediated by SIRT1), impacting both its subcellular localization and cell survival [62]. Importantly, the acetylation of K91 within the RRK motif (R89–R90–K91) has been identified as a critical factor influencing the mitochondrial localization of NME4 [59]. Additionally, two other lysine residues, K45 and K72, along with K91, were shown to play pivotal roles in the acetylation of NME4, regulating its dynamic movement between the nucleus and cytosol [62].

NME4 is also involved in mitophagy, a crucial process for cellular homeostasis, via its binding to cardiolipin, thus participating in its translocation to the outer mitochondrial membrane. Importantly, a point mutation in NME4, R90D, disrupted its interaction with cardiolipin and rendered it ineffective in promoting mitophagy [63]. Then in a separate study, the impact of oxidative stress on NME1 was examined in a mouse radiation-induced fibrosarcoma cell line, suggesting that disulfide crosslinking (C109-C109) of NME1 might serve as a potential mechanism for modifying cellular regulation. Furthermore, a point mutant, C109A, did not undergo cross-linking and exhibited properties like the wild type [64]. In a recent investigation, it was demonstrated that NME1 can undergo CoAlation (a posttranslational modification) through covalent modifications in response to oxidative stress. This enzyme isoform was the primary CoA-binding protein in both cultured cells and rat tissues. Furthermore, the study revealed that during mild oxidative stress, CoA competes for binding at the active site of NME proteins, and the C109A mutant is unable to undergo CoAlation [60].

Of importance, among the list of mammalians coAlated proteins, alpha-tubulin has been also reported to be modulated by NME1 [65, 66]. In this regard, PRUNE1 has been reported to interact with alpha- and beta-tubulins and to positively regulate the MT polymerization rate *in vitro* acting as microtubule-associated proteins (MAPs) in neuronal cells [6]. How PRUNE1 could additionally influence these NME posttranslational modifications, with focus on coAlation, will be of importance especially for the regulation of MT dynamics in human disorders, such as microcephaly [6].

## Nuclear functions of PRUNE1 and NME proteins

Distinct enzymatic functions and subcellular localization have been reported for the members of NME family of proteins (i.e., NME1-10), with some isoforms also showing a nuclear localization [67]. Of interest, posttranslational mechanisms (including deacetylation) are found to be of relevance for the cytoplasm to the nucleus (and *vice versa*) shuttling processes [62]. Indeed, the deacetylation of NME4 occurring within its RRK motif inhibits its typical mitochondrial subcellular localization and is responsible for its nuclear shift [62].

Among the nuclear NME isoforms, NME1 and NME2 were described to possess transcriptional activities acting as both activators and suppressors, thus modulating the expression of cancer-associated genes (including CMYC, PDGFA, TP53, and MMP2) [67].

The interaction between NME2 and DNA (mostly occurring at single-stranded pyrimidine-rich regions) involves a consensus motif consisting of G-rich regions flanking a GAGGT “core” [68]. *Via* these interactions, NME2 may enhance or repress gene transcription, including C-MYC and Vinculin, respectively [67].

Among the genes that are transcriptionally modulated by nuclear NME1 isoform, Bcl-2, cathepsin D, and cyclin D1 are decreased through its association with estrogen response elements [69] or physical interaction with the transcription factor AP1, thus promoting cell cycle arrest and apoptotic death in B lymphocytes [70].

Of interest, NME1, NME5, NME7, and NME8 possess a 3′–5′ exonuclease activity with a potential function in proofreading and DNA repair–related mechanisms [71]. In this regard, decreased expression levels of DNA repair genes (e.g., BRCA1) are reported upon NME1 overexpression in melanoma cells [72]. This further suggests that reduced expression of NME proteins could contribute to the genomic instability that drives cancer progression.

Whether or not PRUNE1 could regulate these nuclear functions of NMEs is a matter of debate. Any pool of required nucleotides can be generally assembled by using the Pi generated by PRUNE1, but this would require the presence of its activity during the DNA synthesis phase of the cell cycle. Recent data (as presented at the NME 2023 conference) are indeed demonstrating an oscillatory protein expression for PRUNE1 during cell cycle, especially during mitosis when DNA synthesis occurs. How this cell cycle dependency of PRUNE1’s function in the context of complex formation with NMEs is of functional relevance has to be further clarified in future experiments.

To date, a nuclear localization of PRUNE1 has been demonstrated by immunostaining in several cell lines and tumor tissues, e.g., MB [7], as also presented at the NME 2023 conference.

Recently an additional indication of new nuclear function of PRUNE1 comes from data presented by Samejima et al. [73]. Indeed, PRUNE1 has been reported to belong to the list of those chromatin-associated proteins during mitotic entry starting the phase preceding the prophase, thus further suggesting a potential nuclear role in modulating gene expression in a cell cycle–dependent manner.

A better understanding of the nuclear functions of NME1, and possibly other NME isoforms, and their interaction with PRUNE1 could provide insights into mechanisms underlying malignant progression in cancer with a peculiar nuclear function.

## Extracellular PRUNE1 and NMEs

Despite the absence of secretion signal peptide sequences, NME proteins are secreted by several tumorigenic cells, and their presence in the extracellular space and in the sera from tumor patients has been reported [74]. The association of extracellular NME proteins with tumor progression is of prognostic significance in several cancer types, including acute myeloid leukemia (AML), BC, NB, and colorectal cancer [75].

Both extracellular NME1 and NME2 proteins were described to have a role in the pre-metastatic niche formation by catalyzing ATP production on the cell surface via their phosphotransferase activity as ATP stimulates purinoreceptors (P2Y and P2X classes) to elicit for example inflammation in the tumor microenvironment (TME). Once activated by ATP, the P2Y1 receptor transactivates VEGFR-2, stimulates mitogenic signaling pathways, and induces nitric oxide, thus supporting the lung pro-metastatic niche formation in a triple-negative breast cancer [76]. Interestingly, recent data showed that another likely secreted enzyme adenosine kinase (ADK) forms a complex with NME proteins and fatty-acid-binding protein 4. Within this complex, the interplay between ADK and NME1 indeed regulates the extracellular ADP and ATP concentration to act on purinergic receptors [77].

Of interest, P2Y1 but not P2X receptors have been reported to be modulated by polyPs, substrates of PRUNE1, in neuronal cells to modulate calcium signaling [78], thus suggesting a potential role for PRUNE1 also in extracellular environment. Furthermore, increased extracellular levels of PRUNE1 were detected in sera collected from NSCLC patients at an early tumorigenic stage (i.e., I–II) as compared to healthy control subjects [4]; thus, PRUNE1 might be considered as a prognostic marker for an early diagnosis of lung cancer. PRUNE1 has been also described to modulate the extracellular environment in triple-negative breast cancer (TNBC) to prepare a metastatic niche in the lung tissue regulating the release of inflammatory cytokines and extracellular vesicles (EVs) from tumorigenic cells leading to the recruitment of immune cells to the TME [187]. Whether or not PRUNE1 with NMEs plays a role in the extracellular environment of tumors is however not clear at this time and needs further studies in the near future.

## Clinical significance of PRUNE1 overexpression and NME mutants in cancer

Several point mutants of NME proteins have been extensively studied to understand their functions and how specific amino acid changes can affect enzyme activity, stability, and cellular functions. The importance of the histidine residue at the active site for phosphoryl transfer reactions was unanimously reported, with a point mutant of H118 showing a complete loss in their catalytic activity [79, 80]. In a study involving a breast carcinoma cell line, it was demonstrated that point mutants P96S and S120G, although retaining their capacity for autophosphorylation, exhibited a loss of protein kinase activity and were unable to effectively suppress metastasis [79, 81–83]. The acid-stable S44 phosphorylation of NME1, rather than its NDP kinase activity, plays a pivotal role in cAMP-mediated signal transduction and is associated with the inhibition of metastasis [84]. Additionally, NME1 has been identified as a negative regulator of STAT3 by blocking its phosphorylation at Y705 [85], while the S44A and S120G mutants were found to be ineffective in inhibiting this phosphorylation, leading to increased expression of fibronectin and matrix metalloproteinase-9 (MMP9) in a human lung adenocarcinoma cell line [86]. In an epithelial human breast cancer cell line, MDA-MB-231, it was observed that when NME1 hexamers undergo oxidation, they disassemble into dimers, resulting in the loss of their metastatic potential and NDPK activity within invasive cells. Furthermore, the C109A mutant exhibits continuous activity as an NDPK and functions as a suppressor of metastasis [87]. It was recently reported that long-chain fatty acyl CoA (LFC-CoA), a metabolic intermediate in fatty acid metabolism, inhibits NME1 and NME2, thereby influencing cancer outcomes. It was shown that LFC-CoA inhibits NDPK activity, where the CoA moiety interacts with R58 but not GDP through NME2 crystal structure (PDB:3bbf), and the R58E mutant had no impact on the NDPK activity of NME2 but granted resistance to the inhibitory effects of LCFA-CoA. Moreover, by using multiple breast cancer cell lines, overexpression of NME1 wild type and R58E mutant significantly suppressed cell migration [88].

With an opposite effect, the overexpression of PRUNE1 has been shown to induce cell motility in MDA 435-H100 cells [1] in the murine transgenic TNBC (MMTV-Prune1-Wnt1) cells and to induce the expression of phosphorylated STAT3 and MMP9 in cocultured murine macrophages, thus enhancing their migration and polarization status toward anti-inflammatory M2 phenotype [8].

Interestingly, NME1 has been reported to participate in phosphatidylinositol 3-kinase (PI3K) signaling by interacting with p110α, the catalytic subunit of PI3K. Additionally, there exists an inverse relationship between NME1 protein expression and Akt phosphorylation, a key indicator of PI3K pathway activation. Furthermore, it was observed that the NME1 protein mutants, specifically P96S, H118F, and S120G, do not result in the deregulation of the PI3K-AKT axis [89]. A study on the role of NME4 as a metastasis suppressor showed that an inactive NME4 mutant and NME4 deletion promoted epithelial-mesenchymal transition (EMT) and increased cell migration and invasion. This data further suggested that low NME4 expression is correlated with aggressive tumors and poor prognosis [90].

Of interest, a positive correlation between PRUNE1 and PI3K-AKT signaling was found in metastatic MB [7] and TNBC [8]. In detail, PRUNE1, through its binding to NME1, enhances SMAD2/3-mediated TGF-β signaling, and represses PTEN, thus leading to activation of AKT and, subsequently, EMT with the characteristic N-cadherin increase and E-cadherin loss [7].

Furthermore, given the wealth of evidence on the role of NME proteins in inhibiting tumor metastasis, a comprehensive analysis was performed using multiple databases (including ONCOMINE, GEPIA, Kaplan–Meier plotter, cBioPortal, and String) to compare mRNA expression levels, patient survival data, and network analysis of NME-related signaling pathways in breast cancer. These analyses unveiled that elevated expression of NME1 and NME2 could potentially serve as a prognostic marker for breast cancer patients with poor prognosis. Additionally, they suggested that *NME3*, *NME5*, and *NME7* might also function as tumor suppressor genes, although further experimental validation is necessary [91].

With regard to breast cancer, the increased expression of PRUNE1 has been positively correlated with advanced nodal status, distant metastasis to the lung, tumor grading, disease progression, and infiltration of M2-antitumorigenic tumor-associated macrophages (TAMs) [1, 8, 92]. Thus, it might be considered a prognostic marker for the identification of metastatic disease in breast cancer.

To summarize, it is essential to emphasize that the precise mechanism through which NME proteins together with PRUNE1 suppress metastasis remains incompletely elucidated. Nonetheless, the aforementioned information indicates that a direct functional association between NME mutations, increased expression of PRUNE1, and cancer progression can be hypothesized at this time.

### Contribution of PRUNE1 and NME proteins in signaling cascades with regard to cancer and metastasis.

#### GTP production and channeling by NME proteins support GTP binding protein activation and regulation by PRUNE1

Many cellular processes are regulated by GTP binding proteins which are active in the GTP-ligated and inactive in the GDP-ligated form. As a general feature, the activation of GTP binding proteins as signaling molecules is transient in nature and often assisted by at least two classes of functional proteins, the so-called guanine nucleotide exchange factors (GEFs) which catalyze the GTP/GDP exchange, thus activation, and GTPase activating proteins (GAPs) which enhance or allow the hydrolysis of GTP, thus the inactivation. In some cases, a third class of regulatory proteins, the so-called guanine nucleotide dissociation inhibitors (GDIs), stabilize the inactive GDP-ligated state. With their canonical NTP/NDP transphosphorylase activity, class I NME proteins are an additional partner in such signaling processes, as they are able to provide the required GTP from ATP and GDP. The replenishment of GTP does not necessarily occur in close proximity to the GTP binding proteins, but there are several examples where GTP channeling into GTP binding proteins occurs by the neighboring localization of class I NME proteins.

With regard to GTP binding protein activation, PRUNE1 was described to bind with alpha- and beta-tubulin, and to act as MAPs, in order to enhance the MT polymerization rate [6]. Both alpha- and beta-tubulin are GTP binding proteins. The irreversible GTP binding to alpha-tubulin is known to affect the structure of MT, whereas the GTP hydrolysis by beta-tubulin modulates their dynamic instability [93]. Of interest, mutations in PRUNE1 affecting its enzymatic DHH or DHHA2 domains (i.e., p.D30N and p.R297W) have been reported to affect the microtubule polymerization rate *in vitro*. This is probably due to alterations in the GTP-GDP switch regulating the MT dynamics. Further studies are required to verify this hypothesis.

#### Heterotrimeric G protein activation

G protein-coupled receptors (GPCRs) and their signal transducers, heterotrimeric G proteins, are another example. The GPCR exerts the GEF function, the G protein α-subunit is the GTP binding protein, the Gβγ dimer is the GDI, and the GAPs are called regulator of G protein signaling (RGS) proteins. Catalyzing the formation of GTP from GDP and ATP, NMEs regulate the amount of GTP available for G-protein activation, thereby indirectly controlling the G-protein signaling [94–96]. NME2, but not NME1, was found as bound to Gβγ dimer and is thus able to replenish GTP locally [97, 98]. In addition, it was found that NME2 is able transfer the phosphate from its intermediately formed N1-phosphohistidine (N1-pHis) on H118 to H266 in the Gβ subunit forming an N3-phosphoshistidine (N3-pHis) [97, 99]. This phosphate retains sufficient energy to potentially generate a GTP molecule, which is most likely facilitated by the dissociation of GDP from the Gα subunit. Rebinding of this newly formed GTP causes activation of the G-protein without involving a GPCR [100, 101]. Of note, also this mechanism appears to be evolutionarily conserved as it was recently detected to regulate axon regeneration in *Caenorhabditis elegans* [102]. Additional data provided evidence that the interacting NME in mammals is a hetero-oligomer formed by NME2 and NME3 with NME3 mediating the interaction with the G protein [54].

Regarding carcinogenesis and metastasis, numerous reviews have extensively discussed the oncogenic potential of various GPCRs [103, 104]. Nevertheless, constitutive activation of the Gα subunits Gα_s_, Gα_q_, Gα_11_, Gα_12_, and Gα_13_ by mutations interfering with their GTPase activity is also associated with different types of cancer and is seen as driver oncogenes based on aberrant signaling [103, 104]. It is therefore tempting to speculate that a drastic increase in the amount of NME2/NME3 hetero-oligomers causing an increase in GPCR-independent G protein activation might lead to aberrant signaling, too, and might offer one explanation why the action of an increasing expression of NME3 in tumor cells is context dependent [105, 106].

As mentioned above, the eye-color mutation of *D. melanogaster*’s PRUNE1 homologue “pn” shows a lethal interaction with the NME protein homologue “awd” Killer of prune (awd^K−pn^) mutation [27, 28]. Of interest, it was postulated that the lethal interaction (between mutated “pn” and “awd”) is due to a neomorphic mutation increasing the ability of Awd^K−pn^ to induce a GTPase activity into a GTP-bound “on” state, thus altering cell proliferation and differentiation processes [29]. At this time, the data obtained in *Drosophila* need further confirmation, at least in mammals.

#### GTP channeling into dynamin and dynamin-like GTP binding proteins as well as other low monomeric GTPases

The three mammalian dynamin isoforms are large monomeric GTP binding proteins with a rather low affinity for GTP, but a rather high GTPase activity. Thus, locally high GTP concentrations have to be achieved and replenished to keep these proteins in action. NME1 and NME2 have been proven to channel these high amounts of GTP into dynamins [107]. Whether this GTP channeling at the cell membranes is achieved by mono-hexamers of either NME1 or NME2 [107] or by heterohexamers [52] is a matter of debate. NME4 exerts a similar GTP channeling activity for the mitochondrial dynamin-like GTP binding protein OPA1. Also, for a second class of mitochondrial dynamin-like GTP binding proteins, the mitofusins, a channeling of GTP by NME isoforms is anticipated. The role of these NME–dynamin interactions for endocytosis of clathrin-coated vesicles, and mitochondrial inner and outer membrane fusion as well as the involvement of GTP channeling by class I NME proteins to their function as metastasis suppressors is reviewed in this issue of *Cancer and Metastasis Reviews* [108] and is therefore not discussed here.

A large variety of monomeric GTPases, also called low molecular weight GTP binding proteins, of the Ras superfamily have been implicated in tumor metastasis [109]. In contrast to dynamins, Ras superfamily GTP binding proteins exert a high affinity for GTP and, in the absence of GAPs, a low intrinsic GTP hydrolysis rate [110]. The use of GTP by Ras-like GTP binding proteins appears to be rather low and there is no apparent need for direct GTP supply. Very early literature indicated a possible direct activation mechanism, involving the transfer of the highly energetic phosphate group from the intermediate phosphohistidine on NME proteins to the GDP bound within inactive Ras-like GTP binding proteins [111, 112]. This is structurally highly unlikely, as both the bound GDP and the phospho-histidine are deeply buried in the nucleotide binding pocket of the GTP binding protein and the NME protein, respectively [113]. To the best of our knowledge, there is no trustworthy evidence that GTP channeling to low molecular weight GTP binding proteins of the Ras superfamily by NME proteins does occur. Further, elucidative data will be needed in the future to dissect this important question.

With regard to monomeric GTPases, PRUNE1 has been reported to interact with ASAP1, a protein with ADP-ribosylation factor (Arf) GAP activity [5]. Since, NME1 was also found to interact with Arf proteins [114], a potential regulation of endocytosis by the PRUNE1/NME1 complex could be also envisioned. With regard to cell transport processes, including endo- and exocytosis, PRUNE1 was reported to colocalize with early and late endosome markers (EEA1, Rab7). It physically interacts with Gsk-3β during Wnt signaling activation [4, 115]. Thus, at this time, further confirmations need to be presented on whether and how PRUNE1 regulates monomeric GTP binding proteins.

#### PRUNE1 and NME1 as modulators of Ras/Raf/ERK signaling

Mutations in genes contributing to the receptor tyrosine kinase (RTK)/Ras/Raf/ERK signaling are commonly seen in many types of cancer and the activation of this pathway is a major component driving tumor cell proliferation [116]. The two paralogs of the scaffold protein of the Kinase suppressor of Ras (KSR1 and KSR2) allow the assembly of Raf/MEK/ERK complexes and can promote as well as inhibit Ras/Raf/ERK signaling, depending on its expression level. If the optimal concentration is exceeded, a scaffold will start to sequester individual components of the cascade and thus disrupt signaling [117]. Interestingly, the inhibition seen at very high levels of KSR1 expression could be overcome by expressing additional components of the MAPK pathway. KSR1 phosphorylation at S392 (mediated by the Cdc25C-associated kinase C-TAK1) promotes its cytoplasmic sequestration [118], while S392 dephosphorylation (by the phosphatase PP2A) results in re-localization to the plasma membrane and signaling [119]. Although representing an unusual phospho-relay from N1-p-His to Ser, phosphorylation of Ser392 might also be possible by NME1 for example in MDA-MB-435 breast carcinoma cells where NME1 overexpression increased KSR phosphorylation and thus decreased p-ERK levels [120]. In agreement with the hypothesis that NME1 is able to inhibit KSR-dependent MAPK activity, silencing of NME1 in HepG2 liver carcinoma cells leads to increased phosphorylation of ERK [22].

Also, PRUNE1 and MAPK are positively correlated in multiple tissue arrays of TNBC patients showing high expression levels of PRUNE1 as well as high levels of phosphorylated ERK1/2 in the nucleus [8]. Up to now, a combined action of PRUNE1 and NMEs in enhancing the activation of ERK1/2 and its downstream signaling has not been reported but might be possible. Future research should address this potential mechanism in cancer progression.

## Tumor microenvironment (TME) and cancer energy metabolism

### The tumor microenvironment (TME)

The TME consists of tumor cells, stromal fibroblasts, endothelial cells, immune cells, secreted factors, and extracellular matrix (ECM) components [121–123]. The complex and dynamic bidirectional interactions of tumorigenic cells with the TME components support cancer cell survival, clonal evolution, local invasion, and metastatic dissemination spread, and shape the therapeutic responses and resistance [121].

Immune cells belonging to both adaptive (T cells, B cells, and natural killer (NK)) and innate (macrophages, neutrophils, and dendritic cells) inflammatory responses are critical components of the TME. Depending on the context, the immune cells can either suppress (pro-inflammatory TME) or promote (immune-suppressive TME) the tumor progression, thus exerting both pro- and anti-tumorigenic functions [124].

The composition of the TME varies between tumor types, and, depending on the immune landscape, three categories have been described: “immune infiltrated,” “immune excluded,” and “immune silent.” The “immune infiltrated” tumors are characterized by immune cells that are uniformly disseminated throughout the TME, thus indicating an active immune response (i.e., “hot” TME). In contrast, those tumors classified as “immune excluded or silent” (i.e., “cold” TME) have not infiltrated immune cells within the TME; and the immune cells are located at the periphery of tumors (“immune excluded”) or absent (“immune silent”).

Among the immune infiltrating cells, there are distinct populations of lymphoid and myeloid cells with different functions in influencing cancer progression. The presence of CD8 + T cells and T helper 1 (Th-1) cells is associated with positive outcomes in cancer patients [125]. In contrast, regulatory T cells (Tregs) suppress the inflammatory anti-tumor immune responses in the TME (via immunosuppressive mechanisms), thus enhancing the tumor progression [126]. A high density of tumor-infiltrating NK cells has been associated with a good prognosis in multiple human solid tumors [127]. B cells exert both anti-tumorigenic functions promoting cytotoxic responses and pro-tumorigenic actions stimulating the acquisition of immunosuppressive phenotypes in the other immune infiltrating cells [128].

Regarding the myeloid cells, both TAMs and neutrophils display plasticity and reprogram their function in different inflammatory contexts and tumorigenic stage. They can be categorized as either proinflammatory (M1 or N2) phenotypes involved in phagocytosis of cells, or immune-suppressive (M2 or N2) phenotypes that promote tumor growth later in tumor development [129, 130]. Furthermore, dendritic cells (DCs) can initiate antitumor immunity. However, once recruited to the TME, DCs are reprogrammed by the secreted cytokines into immune-suppressive/tolerogenic regulatory DCs to support tumor progression [131]. Myeloid-derived suppressor cells (MDSCs) show immunosuppressive functions by suppressing the immune response and shielding tumorigenic cells from the immune attack, thus ultimately stimulating the tumor growth and the local and metastatic dissemination [132].

#### Role of PRUNE1 and NMEs in immune cells and inflammatory response

NME proteins are also found involved in inflammation and immune cell modulation during the immune response. In this regard, NME proteins have the potential to activate inflammatory mechanisms via NLRP3 and caspase 1 activation (independently of TLR4 and pyroptosis) and stimulate IL-1β production in monocytes, thus promoting AML cell survival [133].

Furthermore, NME3 was described as a positive regulator of NF-κB signaling in response to bacterial flagellin, a novel antitumor ligand acting through toll-like receptor 5 (TLR5) and inflammatory pathways (NF-κB) to induce host immunity and aid in the clearance of tumor xenografts [134, 135], thus enhancing the immune cell recruitment into the TME.

A crosstalk between NME proteins and the NF-κB pathway that has been also shown for a long form of NME1 (NME1L) is a splice variant of NME1, containing 25 additional N-terminal amino acids derived from the second exon. NME1L has been reported to specifically bind to IKKβ via its N-terminal region and to inhibit its function. As a consequence, NME1L expression inhibits the phosphorylation and the subsequent degradation of IκB, thus affecting NF-κB activity and its target TNFα. Thus, NME1L, acting as an inhibitor of TNFα-stimulated NF-κB activation, downregulated the expression of TNFα target genes that are involved in cancer cell motility [136].

NME proteins also modulate the inflammatory environment via TGF-β signaling modulation. TGF-β exerts powerful anti-inflammatory functions and is a master regulator of the immune response. NME1 was reported to be physically associated with the serine-threonine kinase receptor-associated protein (STRAP), whose function also consists of stabilizing the association between the TGF-β receptor and Smad7, to negatively influence the TGF-β pathway. NME1-STRAP complex formation requires cysteine residues of both NME1 (Cys145) and STRAP (Cys152 and Cys270), making this complex redox status dependent [137]. These data indicate that the direct interaction of NME1 and STRAP is crucial for the regulation of TGF-β-dependent biological activity, including the modulation of the TME.

Furthermore, NME1 and NME2 were found with distinct roles in B lymphocytes during the immunoglobulin heavy-chain class switch recombination (CSR), a DNA rearrangement occurring when B cells encounter antigens and allowing the switching from the production of IgM to the generation of secondary isotype antibodies (IgG, IgE, or IgA). This process involves DNA double-strand breaks in repetitive DNA elements (called switch S regions) followed by the repair of DNA [138]. Through reverse ChIP proteomic assays, NME2 was found as a protein that binds to double-strand breaks in a B cell line (CH12 cells) stimulated to undergo CSR. Of interest, while the knockdown of NME2 resulted in reduced CSR in the CH12 B cell line, the reduced expression of NME1 increased CSR in the same cell line. Furthermore, while NME2 binds to the S regions only upon CSR stimulation, in contrast NME1 was found to bind S region in unstimulated B cells [139]. These results suggest that NME1 and NME2 proteins have opposite roles in B cell modulation during immune response.

NME2 protein was also found to be required for T lymphocyte activation through the K_Ca_3.1 channels via histidine phosphorylation of H358, stimulating their proliferation and activation [140]. These data indicate a role for histidine phosphorylation in immune cell activation during immune cell response.

The interaction between NME1 and PRUNE1 was shown to affect signaling pathways with a role in the TME. In this regard, a metastatic axis driven by PRUNE1 and NME1 interaction was reported in metastatic MB group 3 where both genes are overexpressed [7]. PRUNE1, through its binding to NME1, impairs its inhibitory activity on the canonical TGF-β pathway, thus enhancing TGF-β signaling, resulting in Orthodenticle homeobox 2 (OTX2) and Zinc finger protein SNAI1 (SNAIL) upregulation, Akt pathway activation via PTEN repression, and EMT in metastatic MB [7].

TGF-β acts as a driver that orchestrates the immunosuppressive TME, acting as an “endocrine” cytokine, thus promoting tumor growth and metastasis [141]. Indeed, the increase in TGF-β signaling based on the NME1/PRUNE1 interaction enhanced the recruitment and polarization of immunosuppressive M2-TAMs and Tregs into the TME of human samples as well as in a murine model of metastatic TNBC, via modulation of cytokine secretion (inducing IL-17F, IL-28, and IL-20) and exosomal protein content (VIM, SDCBP, IFITM3) [8].

#### Energy metabolism in tumorigenic cells and in TME

Cancer cells adapt their metabolism to meet bioenergetic and biosynthetic demands that are required to sustain tumor growth thus facing the dynamic changes in nutrient and oxygen availability that occur within the TME during tumor progression. The tumor growth and the metastatic processes are fueled by increased glycolysis, glutaminolysis, and lipolysis. In this regard, the increased glycolysis occurring in tumors even when oxygen is present is referred to as the “Warburg effect,” where glucose is converted to lactate via aerobic glycolysis. Indeed, glucose uptake and lactate production are often high in tumor tissues, which can leave extracellular levels of glucose reduced and lactate elevated [142]. In addition to the increased requirement for ATP, tumorigenic cells also necessitate an increment in the biosynthesis of macromolecules (lipids, amino acids), reducing equivalents (NADH), and other cofactors for metabolic reactions [143]. Thus, the metabolic plasticity of the tumorigenic cells is driven by a variety of mechanisms that stimulate energy-producing pathways, including glycolysis, the tricarboxylic acid (TCA) cycle, and fatty acid synthesis [144–149].

The TME is metabolically and spatially heterogeneous because of the shuttling of metabolic intermediates between tumor and immune-infiltrating cells competing for limited oxygen and nutrient supply in poorly vascularized or highly metabolically active areas. Furthermore, because of the complex interplay between available nutrients and metabolic activities, the metabolic reprogramming of any cell type in the TME affects the metabolic features of other cells in proximity. In this regard, the reduction of available arginine in the TME due to their increased consumption by tumorigenic cells and M2-TAMs [150, 151] leads to a reduction of mTORC1 activity in T cells and inhibition of immunosurveillance mechanisms [152]. Similarly, the excessive release of kynurenine in the TME (from tryptophan catabolism in tumorigenic cells) promotes the conversion from conventional CD4 + T cells into Tregs [153], and the expression of the immunosuppressive marker PD-1 on CD8 + T cells, thus affecting their cytotoxic action [154]. In turn, this metabolic-mediated immune suppression of T cells within the TME leads to deregulated and fragmented mitochondria, increased production of reactive oxygen species (ROS), and reduced glycolysis, thus impairing T-cell mediated immunosurveillance and supporting immunosuppression [155].

Different metabolic reprogramming of both cancer and tumor-infiltrating immune cells are activated in distinct regions of the TME. Indeed, those tumorigenic cells that are localized in hypoxic districts mainly secrete lactate, whose production results in the generation of an immune suppressive environment mostly characterized by Tregs [156] and M2-TAMs, with increased amount of arginase-1 (ARG1), interleukin-10 (IL-10), and polyamines [157]. In contrast, those cells that are located in less angiogenic tumor zones predominantly utilize glucose as energy fuel, while the central regions of the tumors that are characterized by necrotic events exhibit decreased amounts of several amino acids (including glutamine, arginine, asparagine, serine, and aspartate) compared with the other tumor sites [158, 159]. Thus, the TME heterogenicity is mainly due to a metabolic shift of both tumorigenic and cancer cells.

### Modulation of metabolic enzymes by PRUNE1 and NME1

NME proteins and associated regulatory pathways might indeed contribute to the dramatic reprogramming of the metabolic pathways in the TME. Two metabolic enzymes, ACLY [160] and SCS [161], have been found to be phosphorylated on H760 and H299 by NME1, respectively. Both are substrates for dephosphorylation by PHPT1 [162, 163], and ACLY has also been suggested to be a substrate for LHPP [164]. ACLY is cytosolic and catalyzes the generation of acetyl CoA central to the biosynthesis of fatty acid and cholesterol. ACLY is highly expressed in several types of cancers, and its inhibition has been shown to lead to proliferation arrest in cancer cells [165, 166].

SCS, also known as succinyl CoA ligase (SUCL) or succinate thiokinase, is an enzyme of the citrate cycle. In eukaryotes, the enzyme is a heterodimer, consistent with α subunit (encoded by the SUCLG1 gene) and a β subunit (encoded by either the SUCLG2 or the SUCLA2 gene) [167, 168]. It couples the reversible conversion of succinyl-CoA to succinate allowing for the formation of either GTP or ATP from GDP or ADP, as determined by SUCLG2 and SUCLA2, respectively. In the reverse mode, an intermediate N3-pHis is formed on the catalytic His299 in the α subunit by either ATP or GTP depending on the β subunit. This histidine is also the target of phosphorylation by NME1. The reverse product of the SCS, succinyl-CoA, can affect cellular metabolism by a covalent modification of lysine residues, a process called succinylation [169]. Succinyl-CoA production and consumption therefore contribute to the complex regulation of glucose metabolism and protein function. Also in tumor cells, changes in succinyl-CoA concentration can affect these cells by regulating metabolism directly and by acting as a substrate required for protein succinyl-modification [170, 171].

That both enzymes, ACLY and SCS, as well as their phosphorylation status on histidine residues regulated by NME1 and its counteracting phosphatase, might be important for cancer development and the TME has been recently substantiated in the aforementioned preprint [172].

A third NME-regulated protein, Fructose-1,6-(bis)phosphate aldolase, is an important enzyme in glycolysis. It catalyzes the reversible cleavage of fructose-1,6-(bis)phosphate and fructose 1-phosphate to dihydroxyacetone phosphate and either glyceral-dehyde-3-phosphate or glyceraldehyde, respectively. The neuronal specific isoform aldolase C can be phosphorylated by NME1 on D319 [173]. As stated above, increased glycolysis is a hallmark of tumor cells and thus higher expression of enzymes taking part in glycolysis is found in many types of cancer. It has been shown that NME1 regulates the transcription and expression of aldolase C in melanoma cells [174]. More recent work indicated that aldolase C expression is shaping the malignant phenotype of melanoma cells and their metastatic microenvironment in the brain [175]. These data highlight the importance of NME1 as regulator of aldolase C; However, its role on the formation of the phosphoaspartate on aldose C in the TME remains unknown and needs to be investigated. In summary, by further studying NME-mediated phosphoryl transfer occurring on metabolic proteins, we might be able to dissect additional NME-related functions concurring on metabolic response in physiological conditions.

NME proteins are found as regulators of inflammasome activation also through metabolic pathway modulation. NME4, mainly located on mitochondria, was found among the inflammasome regulators in mouse macrophages through a genome-scale arrayed siRNA screen. NME4 regulates the switch from aerobic to glycolytic metabolism in LPS-induced mouse macrophages, and promotes mitochondrial DNA synthesis and the exposure of cardiolipin phospholipid on the mitochondrial membrane surface, thus acting as a positive modulator of noncanonical and canonical inflammasome activation. Additionally, NME4 is involved in TNF receptor-associated factor 6 (TRAF6) recruitment to mitochondria, thus also stimulating the production of ROS. These data indicate that NME4 stimulates an inflammasome-related response by acting on mitochondrial and metabolic processes [176].

As mentioned before, PRUNE1 exhibits PPX/PPase activity. PolyPs ranging from three to several hundred residues are ubiquitously expressed at micromolar concentration in different subcellular localization levels, including cytoplasm, nucleus, and mitochondria [177], exerting a crucial role in the regulation of energy production [178]. How PRUNE1 contributes to the maintenance of the intracellular polyPs pool and to modulate ATP production is a matter of near future studies especially focusing on the contribution of polyPs (PRUNE1 substrates) and their synthesis by affecting the mitochondrial F1 Fo—ATP synthase pump, as recently demonstrated [179].

## Potential therapeutic approaches to fight metastases via energy metabolism approaches

To date, the exact role of NME proteins in the metastasis progression process remains controversial. However, most studies have demonstrated that the transphosphorylase activity of NME1/2 promotes metastasis suppression. Indeed, several approaches increasing the cellular level of NME with the aim of suppressing the metastatic dissemination in several tumors were chosen. These approaches include adenovirus-associated vectors [180], cell-permeable NME transduction [181], or the use of medroxyprogesterone acetate (MPA) that has been reported to increase NME1 transcription [182]. However, a phase 2 clinical trial performed with MPA treatment in those metastatic hormone receptor–negative breast cancer patients failed [183].

Recently, with screening approaches to identify compounds to enhance the transphosphorylase activity of NMEs, the Nm23 activator 1′ (NMac1) has been discovered. NMac1 is a racemic mixture, and the single stereoisomers (NMac2 and NMac3) have been also reported to exert a similar transphosphorylase activation compared to NMac1. NMac compounds can directly bind to the C-term region of recombinant NME1 protein, and among the key interactions between NMacs and NME1, the methoxy group in the ring A directly connected to cyclohexene ring was predicted to form a hydrogen bond with Q147.I. Of interest, NMac1 was found to slightly affect the hexamer formation of NME1. The biological significance of the increased transphosphorylase activity of NME1 caused by NMac1 treatment was studied in a highly invasive breast cancer cell line, MDA-MB-231 cells (expressing low levels of NME1). Upon NMac1 treatment, MDA-MB-231 cells underwent morphological changes (with reduced ruffles and increased cell-covered area) and showed a decrease in active Rac1-GTP formation. Of importance, the anti-metastatic function of NMac1 was verified *in vivo* by using breast cancer metastasis xenograft orthotopic mouse models. The data showed inhibition of lung metastasis *in vivo*, without affecting the primary tumor size, in those preferentially NMac1-treated mice. Furthermore, the daily oral administration of NMac1 did not show signs of toxicity [184]. These results indicate a potential for NMac1 to inhibit metastasis by augmenting the transphosphorylase activity of NME1. Although these results were promising, no follow-up study by the same authors or others confirmed the therapeutic potential of the NMacs so far.

However, in contrast, in some other tumors (e.g., NB [14] and MB [7]) the high expression of NME1 is correlated with metastatic disease and worse prognosis. One of the pro-metastatic mechanisms postulated for NME proteins relies on their interaction with protein binding partners. Thus, in those tumors that are characterized by an inverse correlation between NME1 levels and poor outcomes, a therapeutic approach impairing the interaction of NME proteins with specific binding partners appears to be promising. Indeed, the use of a synthetic competitive cell-permeable peptide (CPP) with the ability to affect the NME1-PRUNE1 interaction has therapeutic benefits in different tumor models *in vitro* (e.g., breast and colon cancer cells) [185] and *in vivo* (NB and prostate cancer models [185]). CPP mimicked the minimal region of NME1 that is predicted to be phosphorylated by CK1/2 and is responsible for the interaction with PRUNE1 [32]. Thus, this peptide was generated by fusing the amino acid sequences (i.e., amino-acids 115–128) [3] necessary for NME-1–PRUNE1 interaction to the trans-activating protein of the cell-penetrating region of human immunodeficiency virus. To ensure its optimal delivery *in vitro* and *in vivo*, an adenovirus type V encoding CPP in its genome was created. Thus, once the virus is infected, these cells receive copies of CPP which competes with endogenous NME1 for the CK1/2-mediated phosphorylation and the subsequent interaction with PRUNE1. The therapeutic efficacy of CPP in inhibiting the metastatic process *in vivo* was shown for NB using heterotopic xenograft mice injected with SH-SY5Y-Luc cells previously transduced with the adenoviral particles carrying CPP [185] using *in vivo* bioluminescence imaging technology [186].

Similar data were obtained in a xenograft mouse model of prostate cancer providing evidence that CPP reduces prostate cancer metastases formation *in vivo* [185]. Furthermore, the displacement of NME1 from PRUNE1 by CPP also impaired the tumor growth and the metastatic dissemination of MB group 3 *in vivo* in an orthotopic xenograft mouse model generated by implantation of D425-Med-Luc cells transduced with adenoviral particles carrying CPP. Of interest, the inhibition of the TGF-β pathway by inhibition of nuclear SMAD2 and of EMT was reported to occur in the TME of those mice injected with CPP-bearing cells [7].

**Fig. 3:**
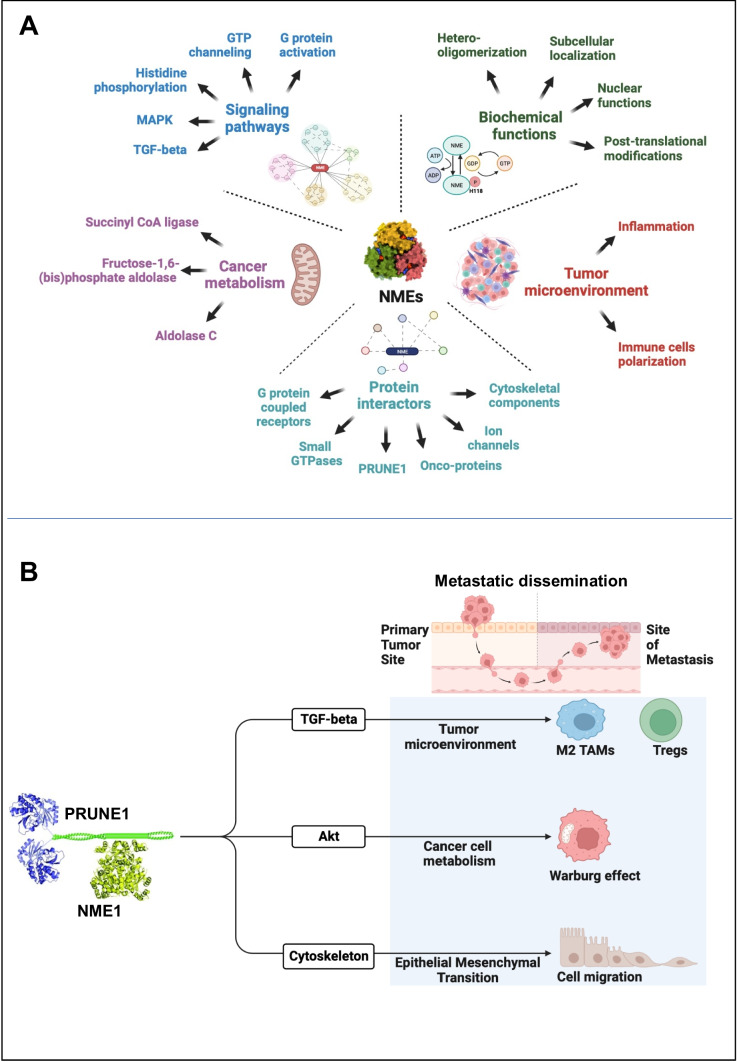
**A** Schematic representation of the general aspects of NME proteins as discussed in this review. The biochemical functions of NMEs in the cells are mostly dictated by their oligomerization status, subcellular localization (including nuclear functionalities), and posttranslational modifications. NME proteins are found to modulate a variety of signaling cascades, including MAPK and TGF-β, through histidine phosphorylation, G protein activation, GTP channeling, and binding to several protein interactors, including G protein-coupled receptors, small GTPases, ion channels, and metastasis-related proteins (e.g., PRUNE1). Altogether, these NME-related pathways result in functions in cancer cell metabolism (via regulation of metabolic proteins, e.g., Succinyl CoA ligase, Frictose-1,6-(bis)phosphate aldolase, and Aldolase C) and in inflammatory processes, thus redirecting the immune cell polarization status within the tumor microenvironment (TME). **B** Schematic depicting the overall processes modulated by PRUNE1–NME1 complex. The complex formation between NME1 (hexamer) and PRUNE1 (dimer) [187] drives pro-metastatic processes. PRUNE1-NME1 complex enhances the canonical TGF-β pathway responsible for the recruitment and polarization of M2-TAMs and Tregs towards immunosuppressive phenotypes in the tumor microenvironment (TME). Furthermore, the positive modulation of AKT pathway due to the complex formation may promote metabolic changes in tumorigenic cells, thus resulting in pro-Warburg effects. Finally, the interaction of both NMEs and PRUNE1 proteins with cytoskeletal components (e.g., microtubules, MT) promotes cell motility processes via epithelial mesenchymal transition (EMT). The figure was created with BioRender.com

The approach of CPP being able to impair the formation of the NME1/PRUNE1 complex therefore holds promises for the treatment of several metastatic tumors [33]. Thus, we believe that future discoveries with the identification of a larger protein–protein network with PRUNE1 as a master interactor protein will have a great impact on the development of new therapeutic strategies. Here we imagine this larger network of protein interactions would act at multiple levels and influence those already known activities of NME1-4 here summarized (see Fig. 3).

In conclusion, the presented class I NME isoforms with intrinsic and extrinsic functions are known to control several signaling pathways that can be deregulated influencing cancer energy metabolism. We hypothesized at this time that these actions, altogether, result in controlling the innate immunity process, and modifying the immune cell reprogramming and these topics will be the issue of future research. At this time, based on the current knowledge, we envision that by “enhancing or lowering” NME’s functions as well as disrupting the interaction with PRUNE1 protein, we might identify new therapeutic options to treat cancer and metastasis dissemination.

## Data Availability

N/A.

## Abbreviations

NB: Neuroblastoma
MB: Medulloblastoma
MTs: Microtubules
EMT: Epithelial–mesenchymal transition
BC: Breast cancer
TNBC: Triple-negative breast cancer
polyPs: Inorganic polyphosphates
PPX/PPase: Exopolyphosphatase/pyrophosphatase
Pi: Phosphate unit
NMR: Nuclear magnetic resonance
IDP: Intrinsically disordered protein
N-term: Amino-terminus
C-term: Carboxyl-terminus
CKI and CKII: Casein kinase I and II
MAPs: Microtubules-associated proteins
AML: Acute myeloid leukemia
TME: Tumor microenvironment
EVs: Extracellular vesicles
PI3K: Phosphatidylinositol 3-kinase
PTEN: Phosphatase and tensin homolog
VEGFR-2: Vascular endothelial growth factor receptor 2
TAMs: Tumor-associated macrophages
GEFs: Guanine nucleotide exchange factors
GAPs: GTPase activating proteins
GDIs: Guanine nucleotide dissociation inhibitors
GPCRs: G protein-coupled receptors
RTK: Receptor tyrosine kinase
ECM: Extracellular matrix
Tregs: Regulatory T cells
DCs: Dendritic cells
MDSCs: Myeloid-derived suppressor cells
STRAP: Serine-threonine kinase receptor-associated protein
CSR: Class switch recombination
OTX2: Orthodenticle homeobox 2
SNAIL: Zinc finger protein SNAI1
TCA: Tricarboxylic acid cycle
ROS: Reactive oxygen species
ARG1: Arginase-1
IL-10: Interleukin-10
SCS: Succinyl CoA ligase SUCL
TRAF6: TNF receptor-associated factor 6
OXPHOS: Oxidative phosphorylation
NMac1: Nm23 activator 1
